# Reciprocal positive selection for weakness - preventing olaparib resistance by inhibiting BRCA2

**DOI:** 10.18632/oncotarget.7883

**Published:** 2016-03-03

**Authors:** Mateusz Rytelewski, Saman Maleki Vareki, Lingegowda S. Mangala, Larissa Romanow, Dahai Jiang, Sunila Pradeep, Christian Rodriguez-Aguayo, Gabriel Lopez-Berestein, Rene Figueredo, Peter J. Ferguson, Mark Vincent, Anil K. Sood, James D. Koropatnick

**Affiliations:** ^1^ Department of Microbiology and Immunology, Western University, London, ON, Canada; ^2^ Department of Oncology, Western University, London, ON, Canada; ^3^ Department of Gynecologic Oncology and Reproductive Medicine, The University of Texas MD Anderson Cancer Center, Houston, TX, USA; ^4^ Department of Experimental Therapeutics, The University of Texas MD Anderson Cancer Center, Houston, TX, USA; ^5^ Center for RNA Interference and Non-coding RNA, The University of Texas MD Anderson Cancer Center, Houston, TX, USA; ^6^ Lawson Health Research Institute, London, ON, Canada; ^7^ Department of Cancer Biology, The University of Texas MD Anderson Cancer Center, Houston, TX, USA

**Keywords:** DNA repair, resistance, BRCA2, PARP1, antisense

## Abstract

Human tumor heterogeneity promotes therapeutic failure by increasing the likelihood of resistant cell subpopulations. The PARP-1 inhibitor olaparib is approved for use in BRCA-mutated ovarian cancers but BRCA2-reversion mutations lead to functional homologous recombination repair (HRR) and olaparib resistance. To overcome that resistance and expand use of PARP1 inhibition to cancers with functional HRR, we developed an antisense strategy to render the majority of tumor cells in a population BRCA2-deficient. We predicted that this strategy would render HRR-proficient tumor cells sensitive to olaparib and prevent emergence of resistance in a tumor cell population heterogeneous for HRR proficiency. We report that BRCA2 downregulation sensitized multiple human tumor cell lines (but not non-cancer human kidney cells) to olaparib and, combined with olaparib, increased aneuploidy and chromosomal translocations in human tumor cells. In a mixed HRR-proficient and HRR-deficient cell population, olaparib monotherapy allowed outgrowth of HRR-proficient cells resistant to subsequent olaparib treatment. Combined BRCA2 inhibition and olaparib treatment prevented selection of HRR-proficient cells and inhibited proliferation of the entire population. Treatment with BRCA2 siRNA and olaparib decreased ovarian xenograft growth in mice more effectively than either treatment alone. *In vivo* use of BRCA2 antisense oligonucleotides may be a viable option to expand clinical use of olaparib and prevent resistance.

## INTRODUCTION

Tumor heterogeneity is a feature of most human cancers and increases the probability that small numbers of resistant cells pre-exist at the start of therapy. This phenomenon has been described in experimental models *in vitro* and has been modelled *in silico* using data from clinical studies [1, 2]. Single nucleus genome sequencing of breast cancer specimens has suggested that no two cancer cells in a tumor are exactly the same [3], highlighting the challenge to effective and long-term cancer treatment.

Anti-cancer therapy imposes powerful selection pressure on the polyclonal and diverse tumor ecosystem. It promotes survival of cells with highest fitness and destroys less fit, more susceptible cells, leading to eventual therapeutic failure: a phenomenon consistent with classical Darwinian evolutionary theory [4]. It is necessary, therefore, to design treatment regimens capable of avoiding Darwinian positive selection. Such treatments would not select for fitness and treatment resistance in a heterogeneous tumor cell population, but would select for reduced fitness and susceptibility to treatment.

PARP1 is an enzyme involved in a variety of cellular processes including DNA repair and replication. The exact mechanisms through which PARP1 contributes to DNA maintenance are not completely clear, but PARP1 mediates single strand break (SSB) DNA repair essential for normal DNA replication [5]. Originally it was thought that if SSBs are left unresolved (due to PARP1 inhibition) they can cause replication fork collapse, resulting in double strand breaks (DSBs) that must be repaired by HRR or error-prone non-homologous end joining (NHEJ) [6]. However, that may not be a complete explanation [7]. PARP1 is also directly involved in the maintenance of stalled replication forks by preventing MRE11-mediated degradation of DNA. When a replication fork is stalled due to base damage or other obstacles that hinder the progression of DNA polymerase, MRE11 acts as an endonuclease which degrades the DNA, causing fork collapse and replication failure. PARP-1 prevents this and maintains replication fork integrity, providing the time necessary for DNA damage to be repaired [8].

Given the role of PARP1 in DNA repair and replication, the PARP1 inhibitor olaparib is synthetically and selectively lethal in cells with HRR defects but does not affect HRR-proficient cells [9–11]. The exact causes of this synthetic lethal relationship are still being explored [7], but it has been proposed that cells without functional HRR are unable to repair the DSBs that result from PARP-1 inhibition (*via* unresolved SSBs), a consequence leading to lethal DNA damage. This ability to spare non-cancerous, HRR-proficient cells was the basis for much of the enthusiasm surrounding PARP1 inhibition and spawned a large effort by the biotechnology industry to identify, test, and market a constellation of PARP1-inhibiting drugs [12]. After several clinical trials with mixed results and an FDA rejection for accelerated drug status, olaparib was approved by the FDA for use in advanced ovarian cancer patients with validated BRCA gene mutations [13]. Another PARP1 inhibitor (veliparib) is currently undergoing Phase III clinical trials as a first-line therapy in combination with chemotherapy for BRCA mutation-positive breast cancer [14].

The same characteristics and circumstances that render PARP1 inhibition so attractive in oncology (selective killing of tumor cells with HRR defects) is also part of what can ultimately lead to loss of effectiveness. The applicability and usefulness of PARP1 inhibitors is limited to treatment of tumors composed predominantly or wholly of HRR-deficient cells: this comprises only a subset of all tumors [15, 16]. Furthermore, selective killing of HRR-deficient cells in a heterogeneous tumor population containing HRR-proficient cells can rapidly lead to the outgrowth of HRR-proficient, resistant clones and therapy failure.

At least five separate PARP1 inhibitor resistance mechanisms have been identified in *in vitro* experiments and in clinical studies, including upregulation of drug efflux pumps that decrease drug concentration inside the cell and 53BP1 mutations that reactivate HRR pathway functionality in BRCA1 deficient cells [17–19]. However, the most striking resistance mechanism is the reported reversion of BRCA2-mutated tumors to functional BRCA2 following olaparib treatment [20]. The implications of this are two-fold: 1) BRCA2 mutation status (and by extension HRR-proficiency) is heterogeneous, even in tumor populations primarily composed of BRCA2-mutated cells and; 2) the selection pressure for HRR proficiency is so great during PARP1 inhibitor treatment that tumor cells with functional HRR have a distinct survival advantage and will eventually overtake the HRR-deficient population.

Emergence of PARP1 inhibitor resistance displays the need for a new combinatorial approach to their application in the clinic. Given the unique relationship between PARP1 and HRR status, it is inevitable that PARP1 inhibition alone will select for subclones in tumor cell populations that are proficient for HRR. We hypothesize, therefore, that resistance based on HRR function can be forestalled or even eliminated by combined therapeutic targeting of PARP1 and BRCA2.

Combining PARP1 inhibition with BRCA2 inhibition may be an avenue to prevent resistance *via* a mechanism we term “reciprocal positive selection for weakness”. In a heterogeneous tumor population, BRCA2 inhibition will select for cells with deficient HRR while concomitant olaparib treatment will eliminate those cells. The reciprocal is also true: olaparib treatment will select for HRR-proficient cells which will then be susceptible to BRCA2 inhibition. We propose that such a strategy will prevent the outgrowth of resistant lesions and extend the time that a tumor is responsive to treatment.

In this study, we show that therapeutic BRCA2 inhibition using BRCA2-targeting antisense oligonucleotides (ASOs) is a promising avenue to prevent resistance to olaparib. BRCA2 ASO treatment sensitized lung, ovarian, and breast tumor cell lines to PARP1 inhibition. Importantly, BRCA2 ASO treatment did not increase the susceptibility of non-cancerous HK-2 kidney cells to PARP1 inhibition. Furthermore, combined BRCA2 ASO and olaparib treatment in a tumor cell population with varying degrees of HRR-proficiency prevented the outgrowth of resistant clones. In addition, we found that combined inhibition of BRCA2 and PARP1 *in vivo* delayed the growth of ovarian cancer tumors. This work provides a rationale for combining BRCA2 ASO and olaparib treatment and extends the applicability of olaparib clinically, which up until now has been used primarily in the context of BRCA1 or 2 mutated ovarian cancers.

## RESULTS

### BRCA2 inhibition overcomes innate olaparib resistance in three lung cancer cell lines

Olaparib has limited efficacy in cancer cells with intact HRR [21]. The majority of lung tumors do not exhibit mutations in BRCA1 or 2 genes (BRCA1 or 2 is mutated in 1.8%-11.2% of cases depending on the data set and tumor type) [22]. Thus, olaparib may have little utility in lung cancer treatment as a single agent, given the relatively low levels of BRCA inactivating mutations. To determine whether BRCA2 inhibition could overcome innate olaparib resistance, we tested control or BRCA2 ASO treatment with olaparib in A549 lung adenocarcinoma and H2052 and 211H mesothelioma cell lines, all BRCA2-proficient.

**Figure 1:**
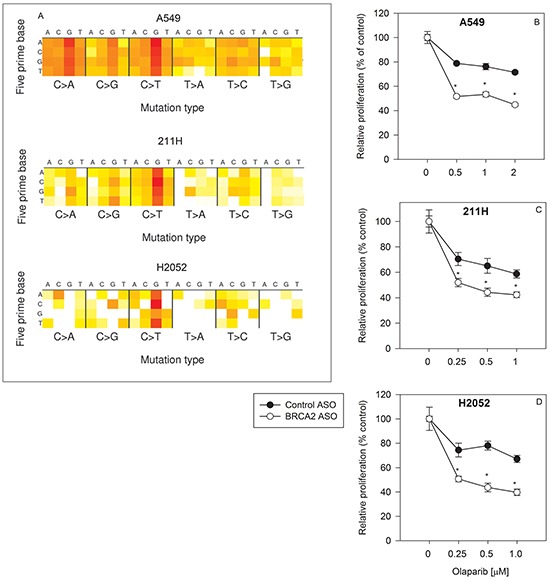
BRCA2 inhibition overcomes innate olaparib resistance in three human lung cancer cell lines A mutation heat map for each cell line was generated using the COSMIC CCLE database interface **A.** A549 **B.**, 211H **C.** and H2052 **D.** cells were transfected with control ASO (●) or BRCA2 ASO (○) and then treated with three different concentrations of olaparib as described in *Supplementary Materials and Methods*. Proliferation was determined by cell counting 96 hours post-transfection (*P<0.05). Means ± SD from representative experiments are shown. All experiments were repeated at least once (N=3).

All three cell lines harbor mutations (513 coding + 616 non-coding mutations in A549 cells; 402 coding + 509 non-coding mutations in 211H cells; and 80 coding + 76 non-coding mutations in H2052 cells) (Figure 1A), suggesting heterogeneity in each population. BRCA2 downregulation increased olaparib sensitivity by as much as 34.5% ± 2.8%, 31.9% ± 8.5%, and 44.1%± 7.8% (p<0.05) in A549, 211H, and H2052 cells, respectively (Figure 1B–1D). BRCA2 ASO treatment sensitized all three lung cancer cell lines to olaparib across the entire range of drug concentrations regardless of mutational signature and load, suggesting that BRCA2 inhibition may render lung tumors with disparate backgrounds sensitive to PARP inhibition.

### BRCA2 inhibition sensitizes ovarian and breast cancer cells to olaparib treatment

Olaparib is approved by the FDA for treatment of BRCA-mutated ovarian cancers [13]. However, only a fraction of ovarian tumors exhibit BRCA1 or 2 mutations [22] and most ovarian cancer patients are not eligible for olaparib treatment. Overcoming innate olaparib resistance in ovarian cancer cells with WT BRCA1 or 2 is potentially valuable clinically.

**Figure 2:**
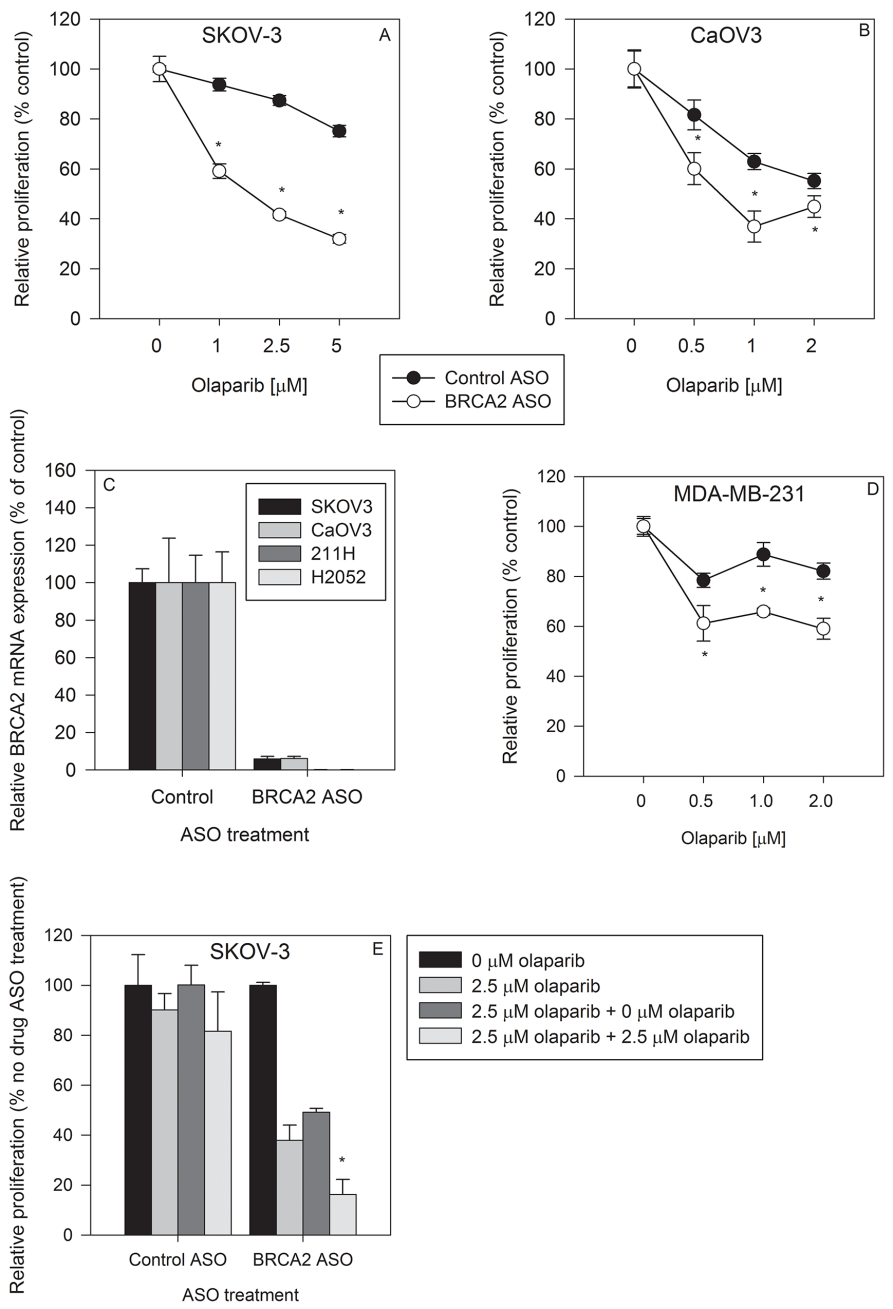
BRCA2 inhibition sensitizes ovarian cancer and breast cancer cell lines to olaparib treatment SKOV-3 **A.** and CaOV3 **B.** cells were transfected with control ASO (●) or BRCA2 ASO (○) and then treated with three different concentrations of olaparib. Proliferation was determined by cell counting 96 hours post-transfection (*P<0.05). **C.** BRCA2 mRNA levels were measured by qPCR 24 hours following BRCA2 ASO transfection in SKOV-3, CaOV3, 211H and H2052 cell lines. **D.** MDA-MB-231 breast cancer cells were transfected with control or BRCA2 ASO, treated with olaparib, and proliferation determined as described above. **E.** SKOV-3 cells were transfected with control or BRCA2 ASO and then treated with olaparib 24 hours post transfection. Ninety-six hours post transfection, cells were counted, plated, and re-transfected with control ASO or BRCA2 ASO and re-treated with olaparib. Cell counts were performed 96 hours post transfection (*P<0.05). Means ± SD from representative experiments are shown. All experiments were repeated at least once (*N*=3).

We tested whether BRCA2 downregulation could sensitize two different ovarian cancer cell lines to olaparib treatment. BRCA2 ASO treatment sensitized SKOV-3 cells to PARP1 inhibition by as much as 52.3% ± 2.7% (p<0.05) (Figure 2A) and CaOv3 cells by 41.3% ± 9.9% (p<0.05) (Figure 2B). The amount of antisense-mediated BRCA2 mRNA knockdown was greater than 90% in both cell lines, similar to the amount of BRCA2 reduction in H2052 and 211H mesothelioma cells (Figure 2C).

Triple-negative (estrogen receptor (ER), progesterone receptor (PR), and Her2/neu [3]) MDA-MB-231 cells were rendered as much as 28.0% ± 5.1% (p<0.05) more sensitive to olaparib compared to cells treated with control ASO (Figure 2D). Therapy options for triple-negative breast cancer are limited [23] and decreasing innate olaparib resistance by BRCA2 downregulation could reveal a new path to more effective treatment.

To determine whether cells that survived the initial BRCA2 ASO and olaparib treatment remain sensitive to subsequent treatment, we treated the same cells with BRCA2 ASO and olaparib a second time. Single BRCA2 ASO + olaparib treatment hindered the proliferation of cells which were re-seeded without any additional olaparib treatment. In addition, a second round of BRCA2 ASO + olaparib treatment decreased SKOV-3 cell proliferation by 67.0% ± 12.4% (p<0.05) compared to cells which did not receive this second olaparib treatment (Figure 2E). This suggests that cells which survive the first round of BRCA2 ASO + olaparib treatment are still sensitive to a second treatment that decreases their proliferation.

### BRCA2 inhibition does not sensitize non-cancerous cells to olaparib treatment

**Figure 3:**
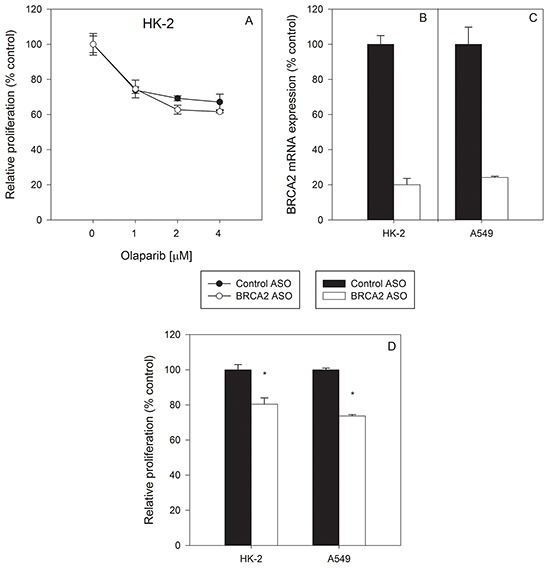
Non-cancerous HK-2 kidney cells werenot sensitized to olaparib by BRCA2 downregulation Non-tumor HK-2 kidney proximal tubule epithelial cells **A.** were transfected with control or BRCA2 ASO and then treated with three different concentrations of olaparib. Proliferation was determined by cell counting 96 hours post-transfection (*P<0.05). BRCA2 mRNA levels were measured by qPCR in **B.** HK-2 and **C.** A549 cells following transfection of BRCA2 ASO. **D.** Changes in proliferation of HK-2 and A549 cells were measured 96 hours post-transfection of BRCA2 ASO. Means ± SD from representative experiments are shown. All experiments were repeated at least once (*N*=3).

An important question when inhibiting BRCA2 in the context of olaparib treatment is whether non-cancer cells are affected to the same degree as tumor cells. Non-cancer, BRCA2-positive HK-2 kidney proximal tubule epithelial cells were treated with either control ASO or BRCA2 ASO followed by olaparib. BRCA2 inhibition did not sensitize HK-2 cells to olaparib at the tested concentrations (Figure 3A). BRCA2 mRNA downregulation was confirmed by qPCR to ensure that lack of sensitization was not due to inadequate transfection (Figure 3B), and the level of BRCA2 mRNA knockdown was similar to that observed in A549 lung cancer cells (which are sensitized to olabarib by BRCA2 ASO treatment) (Figure 3C). In addition, BRCA2 ASO treatment alone induced a minimal but significant reduction in cell proliferation (~20%) in both non-tumor HK-2 and A549 tumor cells (Figure 3D), but no potentiation of olaparib-mediated inhibition of proliferation in HK-2 cells was observed.

### BRCA2 ASO and olaparib treatment induces chromosome aberrations in ovarian and breast cancer cells

Failure of the spindle assembly checkpoint (SAC) results in abnormal chromosomal segregation and can lead to fatal chromosome gain or loss in daughter cells [24]. Both BRCA2 and PARP1 support SAC in mitotic cells [25] and we hypothesized that the decreased proliferation following inhibition of both targets may be due to perturbation of SAC.

**Figure 4:**
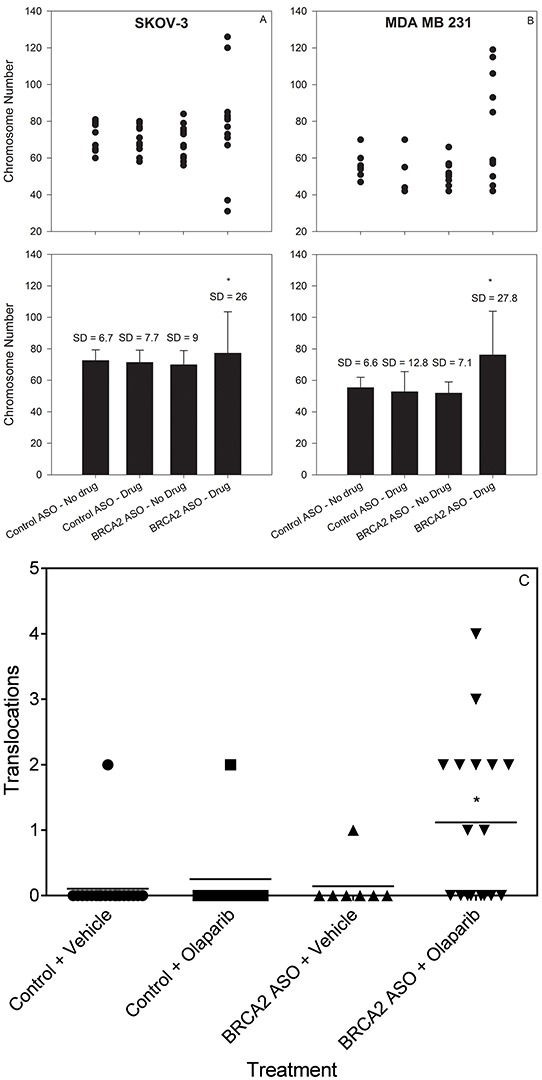
Combined BRCA2 ASO and olaparib treatment increases the variability in chromosome number and increases translocation frequency in ovarian and breast cancer cells SKOV-3 **A.** and MDA-MB-231 **B.** cells were treated with control ASO or BRCA2 ASO in the presence or absence of olaparib. Forty-eight hours following olaparib treatment, cells were fixed and processed to yield metaphase spreads. The number of chromosomes in individual metaphase cells is shown (●). **A', B'**: Mean chromosome number ± SD after each treatment, calculated from the data shown in *panels A and B* (*Difference in SD, *P*<0.05, Bartlett's Test). SKOV-3 **C.** SKOV-3 cells were transfected with control ASO alone or with olaparib or BRCA2 ASO alone or with olaparib. Forty-eight hours post-olaparib, cells were processed to yield metaphase spreads. FISH was performed for chromosomes X, 3, and 16. The number of translocation events in these chromosomes was counted and graphed. Mean numbers of translocations for each treatment are shown as bars (−).*Mean number of translocations were significantly different (*P*<0.05, Welch's t-test). Data from representative experiments are shown. All experiments were repeated at least once (*N*≥10).

We investigated the effect of BRCA2 ASO and olaparib treatment on bulk chromosome number using metaphase spreads of SKOV-3 and MDA-MB-231 cells. Twenty-four hours following olaparib or vehicle treatment, we identified a significant increase in the variance of the chromosome number in cells treated with BRCA2 ASO + olaparib (Figure 4A&4B). This is consistent with the hypothesis that combined BRCA2 and PARP1 inhibition negatively affects the SAC and allows for the mis-segregation of chromosomes, leading to altered aneuploidy in daughter cells.

To determine whether BRCA2 ASO and olaparib treatment had an effect on genome stability we used whole chromosome fluorescence *in situ* hybridization (FISH) probes to label chromosomes X, 3, and 16, and quantify the incidence of random translocations following treatment. Combined BRCA2 ASO + olaparib treatment led to 1.18 mean translocations per metaphase, compared to 0.1, 0.25 and 0.14 for other treatments (*p<0.05) (Figure 4C).

### Combined BRCA2 ASO and olaparib treatment can prevent resistance in a mixed cell line model with varying degrees of HRR

Human tumors exhibit a high degree of heterogeneity [2, 26, 27] which can lead to olaparib resistance [19]. Resistance can occur through a variety of mechanisms [18] including reversion to HRR-proficiency in tumors that were predominantly HRR-deficient prior to treatment [19, 20]. Due to the functional linkage between BRCA2 and PARP-1, we hypothesized that combined BRCA2 ASO and PARP inhibition would prevent reversion to HRR proficiency and the appearance of olaparib resistance.

To test this hypothesis, we used three human tumor cell lines with varying degrees of HRR proficiency: SKOV-3 (BRCA2 WT [28]), MCF-7 (HRR deficient [29]), and CAPAN-1 (BRCA2 mutant [28]).

**Figure 5:**
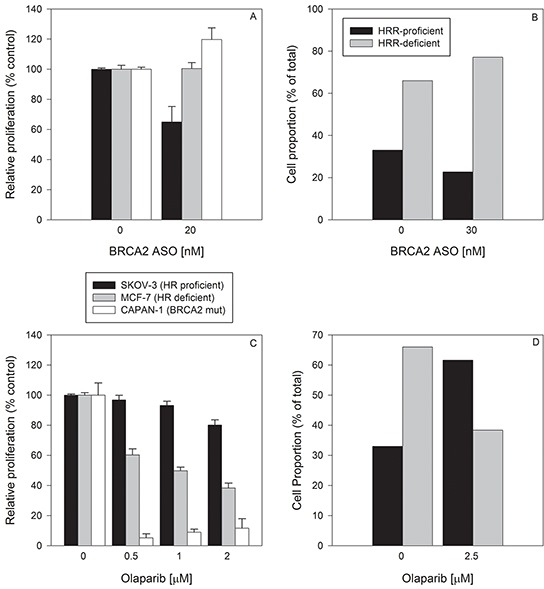
Single treatment with BRCA2 ASO or olaparib has the potential to select for HRR-deficient or HRR-proficient cells, respectively **A.** BRCA2-wild type SKOV-3 cells (*black bars*), HRR-deficient MCF-7 cells (*white bars*), and BRCA2-mutated CAPAN-1 cells (*grey bars*) were treated simultaneously but separately with BRCA2 ASO (20 nM). Due to differing growth medium requirements and to avoid fluorescent label-induced changes in drug sensitivity, cells were not co-cultured. They were treated independently, at the same time with the same materials. Ninety-six hours post-transfection, cells were counted and proliferation determined (% of proliferation after control ASO treatment). **B.** The theoretical proportions of a mixed cell population (HRR-proficient SKOV-3 + MCF-7, and HRR-deficient CAPAN-1) following BRCA2 ASO treatment were calculated using the experimental data shown in *panel A*. **C.** SKOV-3, MCF-7 and CAPAN-1 cells were treated with two different concentrations of olaparib for 96 hours. After drug treatment they were counted and proliferation was determined as a percent of that of vehicle-treated cells. **D.** The theoretical proportions of a mixed cell population (HRR-proficient SKOV-3 + MCF-7, and HRR-deficient CAPAN-1) following BRCA2 ASO treatment were calculated based on the experimental data shown in *panel C*. Data from representative experiments are shown. All experiments were repeated at least once (*N*=3).

When these three cell lines were treated with BRCA2 ASO (20 nM), the proliferation of HRR-proficient SKOV-3 cells was decreased by 35% ± 10% (p<0.05) (Figure 5A), whereas it had no effect in HRR-deficient MCF-7 and CAPAN-1 cells. These data suggest that BRCA2 downregulation in a mixed population of HRR-proficient and HRR-deficient cells would lead to an increased fraction of HRR-deficient, BRCA2 ASO-resistant cells (Figure 5A). When the cell fraction of a theoretical mixed population was calculated on the basis of relative proliferation after treatment with BRCA2 ASO, HRR-deficient MCF-7 and CAPAN-1 cells increased in proportion from a total of 66% to 77% relative to SKOV-3 cells (Figure 5B). Thus, BRCA2 downregulation can select for HRR-deficient cells.

In contrast, a single treatment of each of the 3 cell lines with olaparib decreased the proliferation of HRR-deficient MCF-7 and CAPAN-1 cells by 39% ± 6.8% and 94% ± 4.6% (p<0.05), respectively, but had no effect on SKOV-3 proliferation (Figure 5C). Therefore, the fraction of HRR-proficient SKOV-3 cells in a theoretical mixed population after olaparib treatment increased from 33% to 61% (Figure 5D). Thus, a single olaparib treatment can select for HRR-proficient cells, which is the reciprocal of the effect of BRCA2 ASO.

**Figure 6:**
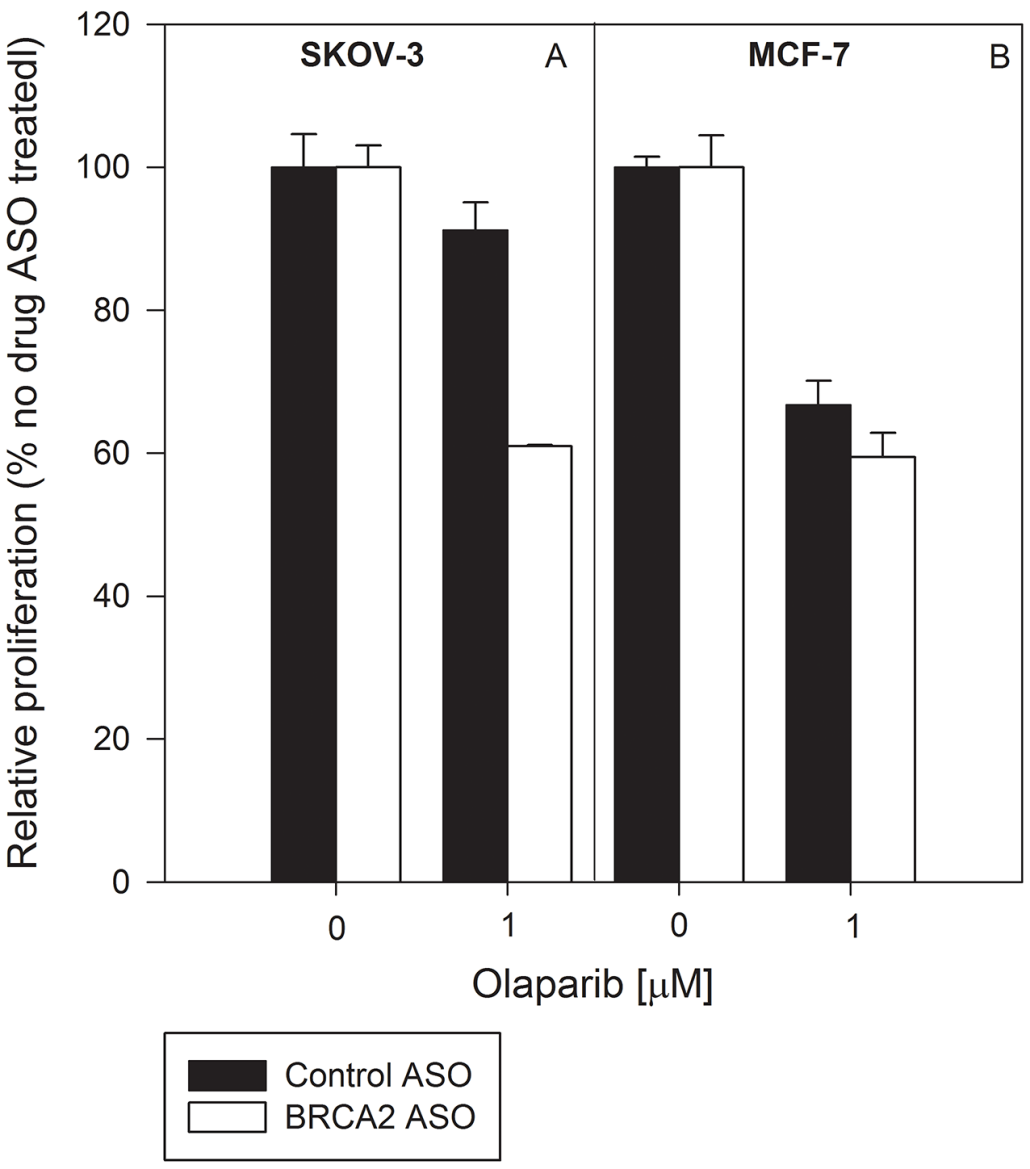
Combined BRCA2 ASO and olaparib treatment decreases the proliferation of both HRR-deficient and HRR-proficient cells BRCA2-wild type SKOV-3 cells **A.** and HRR-deficient MCF-7 cells **B.** were transfected with control ASO (*black bars*) or BRCA2 ASO (*white bars*) and treated with vehicle or olaparib (1 μM). Proliferation was determined using cell counting 96 hours post-transfection. Data from representative experiments are shown. All experiments were repeated at least once (*N*=3).

HRR-proficient SKOV-3 cells (Figure 6A) are resistant to olaparib relative to HRR-deficient MCF-7 cells (Figure 6B). Combined treatment with BRCA2 ASO and olaparib abolished that relative resistance and led to a decrease in proliferation in both cell lines of 40% (p<0.05) (Figure 6A & 6B). This suggests that simultaneous inhibition of BRCA2 and PARP-1 in heterogeneous tumor populations can prevent selection events and forestall emergence of treatment-resistant clones.

### Combined BRCA2 ASO and olaparib treatment can prevent the outgrowth of resistant clones in a co-culture model of BRCA2 heterogeneity

To evaluate the effects of BRCA2 ASO and olaparib on population dynamics and resistance to treatment over time, we devised a co-culture model of SKOV-3 ovarian cancer cells stably expressing either shRNA targeting BRCA2 or control shRNA. This emulated a tumor population with different proportions of cells of varying HRR-proficiency.

**Figure 7:**
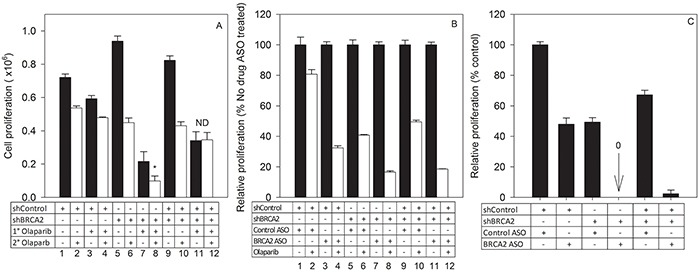
Combined BRCA2 ASO and olaparib treatment prevents outgrowth of resistant cells in a tumor cell population heterogeneous for HRR-proficiency **A.** SKOV-3^shBRCA2^ cells (low BRCA2) were mixed with SKOV-3^shControl^ cells (high BRCA2) at a 3:1 ratio, resulting in a primarily HRR-deficient mixed cell population. Parental and mixed populations were treated for the first time with olaparib (1° olaparib, 2.5 μM) or vehicle. Cells were re-plated at equal density 96 hours post-treatment. Parental and mixed populations were then treated a second time with olaparib (2° olaparib, 2.5 μM) or control vehicle. Ninety-six hours post-treatment, proliferation for all groups was determined based on cell counts and seeding density following 1° olaparib or vehicle treatment. *White bars*: 2° olaparib. *Black bars*: no 2° olaparib. **B.** SKOV-3^shBRCA2^ cells (low BRCA2), SKOV-3^shControl^ cells (high BRCA2), and a mixed cell population (3:1, low BRCA2:high BRCA2) were transfected with control ASO or BRCA2 ASO followed by treatment with vehicle or olaparib (2.5 μM). Proliferation (percent of control ASO-treated cells) was determined 96 hours post-transfection. *White bars*: 2° olaparib. *Black bars*: no 2° olaparib. **C.** SKOV-3^shBRCA2^ cells (low BRCA2), SKOV-3^shControl^ cells (high BRCA2), and a mixed cell population (3:1, low BRCA2:high BRCA2) previously treated with ASO (control or BRCA2) and olaparib (2.5 μM) were re-plated at the same density and allowed to proliferate without further treatment. Data pooled from two independent experiments (*N*=6).

To mimic a heterogeneous tumor cell population that is predominantly HRR-deficient, we co-cultured SKOV-3^shBRCA2^ (low BRCA2) and SKOV-3^shcontrol^ (high BRCA2) in a 3:1 ratio. The mixed cell population, along with unmixed SKOV-3^shBRCA2^ and SKOV-3^shcontrol^ populations, was treated with olaparib (1° Olaparib), then counted, re-seeded and treated with olaparib a second time (2° Olaparib) (Supplementary Figure S2). The mixed cell population, though sensitive to initial treatment with olaparib (Bar 9 vs 10), was completely unresponsive to a second treatment (Bar 11 vs 12) (Figure 7A). The unmixed SKOV-3^shBRCA2^ population remained sensitive to olaparib even after two treatments (Bar 7 vs 8) (Figure 7A). This suggests that 1° olaparib treatment of the mixed cell population selected for HRR-proficient cells and allowed them to outgrow HRR-deficient cells.

To determine if combined BRCA2 ASO and olaparib treatment could prevent the development of resistance among mixed SKOV-3^shBRCA2^ and SKOV-3^shControl^ cells, the mixed and unmixed cells were treated with either control ASO or BRCA2 ASO, in the presence or absence of drug treatment. BRCA2 ASO treatment sensitized the mixed cell population to olaparib (Bar 11 and 12), and the proliferation level of the mixed population following BRCA2 ASO and olaparib treatment was similar to that of the SKOV3^shBRCA2^ cells treated in the same manner (Bar 7 and 8) (Figure 7B).

When mixed and unmixed SKOV-3 populations treated with olaparib and either control ASO or BRCA2 ASO were re-seeded without any further treatment, the mixed cell population that had received BRCA2 ASO + olaparib was unable to proliferate (Figure 7C). This suggests that simultaneous inhibition of both BRCA2 and PARP1 can prevent the outgrowth of resistant cells in a tumor population with HHR heterogeneity.

### Combined inhibition of BRCA2 and PARP1 prevents ovarian tumor growth *in vivo*

**Figure 8:**
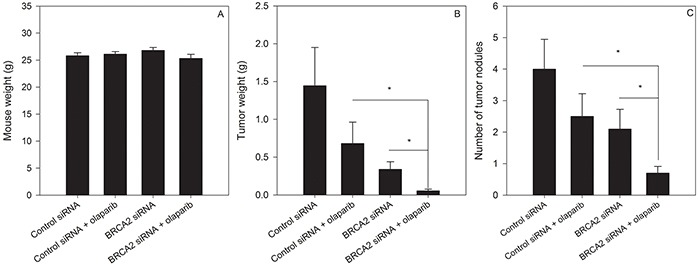
BRCA2 inhibition sensitizes ovarian cancer tumours to olaparib treatment *in vivo* Female athymic nude mice were injected with 1.0×10^6^ SKOV3-IP1 cells *i.p*. Mice were treated 7 days later with olaparib (5 mg/kg 5 days a week *i.p.*) and either control or BRCA2 siRNA twice per week encapsulated in DOPC-liposomes (150 μg/kg) (*N*=40, 10 animals per group). Once the mice in any group were moribund, the animals were weighed **A.** and euthanized. The tumour weight **B.** and number of tumour nodules **C.** were determined (P*<0.05, Student's *t*-test).

We determined whether combined BRCA2 and PARP1 inhibition could prevent or delay growth of ovarian tumors *in vivo*. Female athymic nude mice were injected with SKOV3-IP1 cells i.p and treated 7 days later with control siRNA or BRCA2 siRNA in the presence or absence of olaparib. Following 7 weeks of treatment, mice were weighed (Figure 8A), euthanized and dissected to determine the number and combined weight of tumor nodules in the peritoneal cavity. BRCA2 siRNA + olaparib treatment decreased both the number (Figure 8B) and weight (Figure 8C) of tumors relative to BRCA2 siRNA or olaparib treatment alone (p<0.05), suggesting that combing BRCA2 reduction with PARP1 inhibition may be useful to decrease tumor burden.

## DISCUSSION

The PARP1 inhibitor olaparib is approved for treatment of BRCA-mutated ovarian tumors. However, this represents only a subset of cancer patients [13] and resistance can occur even in this population [20]. We propose a new therapeutic strategy to inhibit BRCA2 function using a BRCA2-targeting ASO, in an effort to overcome these challenges to PARP inhibition in the clinic. In this study, we tested a BRCA2 ASO in combination with olaparib to determine whether the combination could: 1) overcome innate resistance and increase the potential usefulness of olaparib by rendering HRR-proficient, BRCA2-positive tumors sensitive to the drug, and 2) prevent acquired resistance in cell populations with mixed HRR-proficiency.

We show, using human lung, ovarian, and breast cancer cell lines, that BRCA2 ASO treatment can overcome innate resistance to olaparib in these cell lines. None are reported to harbour BRCA2 or BRCA1 mutations (COSMIC CCLE database) and, with functional BRCA2 capable of mediating HRR, are relatively resistant to the therapeutic effects of PARP1/2 inhibition by olaparib. Therefore, BRCA2 ASO treatment has the potential to render a high proportion of tumor cells sensitive to olaparib treatment, which may extend the usefulness and applicability of this drug in the clinic.

The fact that olaparib primarily targets HRR-deficient tumors is also a potential problem due to positive selection for resistant clones in a heterogeneous tumor ecosystem. Most tumors exhibit complex polyclonal variability and data from single nucleus sequencing of breast tumors suggests that no two tumor cells are identical [3]. This renders resistance to targeted therapy and chemotherapy inevitable mathematically [1], and very common biologically [26, 30]. Several olaparib resistance mechanisms have already been described, including the outgrowth of tumors with re-activation mutations in BRCA2 which render olaparib ineffective [18]. In addition, BRCA1-mutated tumors cells with a concomitant mutation in 53BP1 are no longer HRR-deficient and also exhibit resistance to PARP1 inhibition [31]. It therefore appears that olaparib treatment will fail at high frequency without any corresponding positive selection pressure for cells with HRR-deficiency.

Combining BRCA2 inhibition with PARP1 inhibition can achieve a state where each individual treatment positively selects for cells with unique susceptibility to the other treatment, thus preventing or delaying resistance: this is the essence of the concept which we have termed reciprocal positive selection for weakness. The data from our mixed cell experiments suggests that simultaneous inhibition of BRCA2 and olaparib treatment has the ability to limit the proliferation of tumor cells heterogeneous for HRR-proficiency, thus preventing positive selection of resistant cells based on ability to repair DNA.

An important consideration is whether it is possible to develop resistance to simultaneous BRCA2 and PARP1 inhibition (either through a primary mechanism related to HRR, or a secondary mechanism unrelated to HRR proficiency). It may be possible to address this question using a barcoded shRNA library to downregulate an assortment of genes in the context of BRCA2 deficiency and olaparib treatment. The shRNA barcode could be used to determine which gene or genes were down-regulated in any surviving cells. This experiment would divulge whether resistance to combined BRCA2 and PARP1 inhibition is possible, and if so, identify a subset of targets for further study and promote development of strategies to prevent or overcome this potential resistance mechanism.

Our *in vivo* data suggest that it is possible to combine BRCA2 inhibition and olaparib treatment to reduce tumor burden in animals. The mice which received combination treatment exhibited the fewest tumor nodules, and the lowest tumor weight relative to control and each of the single treatments. In particular, the i.p model recapitulates several hallmarks of later stage ovarian cancer, and is a model to explore the potential of therapy to prevent the establishment of metastatic lesions at secondary sites in the peritoneal cavity [32]. Our data suggests that a potential therapeutic outcome of combined BRCA2 downregulation and olaparib treatment would be to prevent tumor spread and growth at secondary sites in the peritoneal cavity following surgical resection of primary tumors. However, further experiments are necessary to determine the effect of BRCA2 inhibition and olaparib treatment on survival of tumor-bearing animals. In addition, to expand the *in vitro* data showing lack of olaparib sensitization in non-cancer HK-2 cells, an *in vivo* study using an siRNA targeting the mouse sequence of BRCA2 will be necessary to elucidate the potential effects of BRCA2 inhibition on olaparib sensitivity in normal tissues.

## MATERIALS AND METHODS

### Cell lines

All cell lines were obtained from the ATCC and maintained at standard conditions (37°C, 5% CO_2_) in AMEM or DMEM medium supplemented with 10% FBS unless otherwise noted. Cell culture medium, serum, reagents and plasticware were purchased from Wisent Inc. (Mississauga, Canada), Life Technologies ThermoFisher, Inc. (Burlington, Canada), and VWR Canlab (Mississauga, Canada). CAPAN-1 cells (ATCC) were grown in Iscove's medium supplemented with 20% FBS. HK-2 cells (ATCC) were grown in Keratinocyte Serum Free medium supplemented with 0.05 mg/ml bovine pituitary extract and 5 ng/ml human recombinant epidermal growth factor.

### Cell proliferation assay

Four hours post-transfection, cells were seeded into 6 well dishes in appropriate experimental groups. Twenty-four hours post transfection, cells were treated with olaparib (Selleckchem, Houston, TX) at three concentrations. Ninety-six hours post transfection, cells were collected and counted (Coulter Particle Counter). Proliferation was determined based on initial cell density and calculated as a percentage of ASO + vehicle treated cells.

### ASO transfection

BRCA2 and control ASOs were transfected as described previously [33]. In brief, 1.5×10^5^ cells were plated into each 25 cm^2^ flask. Twenty-four hours following plating, the cells were transfected with 20nM control or BRCA2 ASO using lipofectamine 2000 (Invitrogen). Four hours post transfection fresh medium was added to the flasks, or the cells were trypsinized and collected for further use.

### Generation of cells stably expressing shBRCA2 and shControl

SKOV-3 cells (ATCC) were transfected with linearized plasmids containing the shControl and shBRCA2 constructs (Cat. No. 336312 Qiagen) using Lipofectamine 2000 (Invitrogen) according to manufacturer's instruction. Seventy-two hours post transfection, the cells were treated with hygromycin for seven days. Individual colonies were isolated using glass isolation rings and then expanded. Sensitivity to olaparib was tested using cell counting proliferation assays.

### mRNA level quantification

Total mRNA was extracted from cells 24 hours post-transfection using an mRNA extraction kit (Qiagen, Toronto, Canada). Total mRNA was reverse transcribed (M-MLV-RT) into cDNA and the cDNA was used as a template for RT-qPCR. A GAPDH and custom BRCA2 primer and probe set (Life Technologies) was used to perform RT-qPCR along with Taqman reagents (Applied Biosystems – Life Technologies) and the Viia7 (Life Technologies) qPCR machine. A standard curve was used to infer target mRNA levels in the samples.

### Metaphase spread preparation and chromosome counting

Four hours post transfection, cells were collected and plated on microwell containing glass slides. Drug treatment commenced 24 hours post transfection and the cells were allowed to grow for a further 24 hours. Colcemid (Sigma-Aldrich, Oakville, Canada) was added to the medium for the last 2 hours of culture. The cells were washed with PBS and then treated with pre-warmed (37°C) KCL (75 mM) for 20 minutes. Cold (4°C) fixative (3:1 methanol acetic acid solution) was added to the cells for 2 minutes. The fixative solution was then replaced with fresh solution and incubated for a further 20 minutes. The fixative solution was replaced a final time for another 20 minutes and the slide was allowed to air-dry at room temperature. The slides were then mounted with mounting medium containing DAPI or used for subsequent FISH. DAPI mounted slides were used to determine chromosome number using an inverted fluorescent microscope (Olympus, Japan).

### Fluorescence in situ hybridization (FISH) for whole chromosomes

Following metaphase preparation, the slides were washed in 2X SSC (pH 7.0) for 2 minutes at 73°C. The slides were transferred into a 0.005% pepsin solution for 10 minutes at 37°C and then washed with PBS for 5 minutes at room temperature. The slides were fixed in 1% formaldehyde for 5 minutes at room temperature and then washed with PBS. The slides were sequentially dehydrated by immersion in 70%, 85% and 100% ethanol. Diluted FISH probes (Empire Genomics, Buffalo, NY) were applied to the slide, covered with glass coverslips and sealed with rubber cement. The probes and chromosomal DNA were co-denatured on a hot plate at 68°C for 5 minutes. Hybridization was performed at 43°C in a humidified chamber for 4 hours. Following hybridization, the slides were washed with 2X SSC +0.1% Igepal (Sigma) at room temperature to remove the cover slips and rubber cement. The slides were washed at 65°C with 0.4X SSC + 0.3% Igepal for five minutes, rinsed briefly with ddH_2_0 and then air dried. The slides were mounted with DAPI mounting medium and visualized using an inverted fluorescent microscope.

### *In vivo* tumor model

Eight to twelve week old female athymic nude mice were purchased from the National Cancer Institute (Frederick, MD). All mouse studies were approved by the MD Anderson Cancer Center Institutional Animal Care and Use Committee. SKOV3ip1 ovarian cancer cells (1.0 × 10^6^) were trypsinized, suspended in 200 μl of Hanks balanced salt solution (HBSS; Gibco, Carlsbad, CA) and injected into the intraperitoneal cavity (i.p.). Seven days after cell injection, mice were randomly divided into 4 groups: 1) Control siRNA/DOPC 2) BRCA2 siRNA/DOPC 3) Control siRNA/DOPC + olaparib 4) BRCA2 siRNA/DOPC + olaparib (n=10 mice per group). siRNA/DOPC nanoparticles were injected twice weekly (150 μg/kg body weight) and olaparib (5 mg/kg body weight; 5 days a week) (i.p.). Mice were monitored daily for adverse effects of therapy and were euthanized 6-7 weeks after cell injection. At the time of euthanasia, mouse and tumor weight was recorded. Tumor tissue was harvested and either fixed in formalin for paraffin embedding, or frozen in optimum cutting temperature medium (OCT; Miles, Inc., Elkhart, IN) to prepare frozen slides, or snap-frozen in liquid nitrogen for lysate preparation. The individuals who performed the necropsies, tumor collections, and tissue processing were blinded to the treatment group assignments.

### Statistical analysis

The cut off for statistical significance was set as p<0.05 *a priori* for all statistical tests. Student's T-tests were used to evaluate the null hypothesis that there was no difference between means if the data had equal variance and a normal distribution. The data was evaluated for normality and equal variance before using ANOVA in the case of multiple comparisons.

## SUPPLEMENTARY FIGURES


