# Supplementary material for: Reciprocal positive selection for weakness - preventing olaparib resistance by inhibiting BRCA2
Source: Oncotarget. 2016 Mar 3;7(15):20825–39. doi: 10.18632/oncotarget.7883 (PMC4991495; doi:10.18632/oncotarget.7883)
Supplement: Supplementary file 1 [file oncotarget-07-20825-s001.pdf]

SUPPLEMENTARY FIGURES

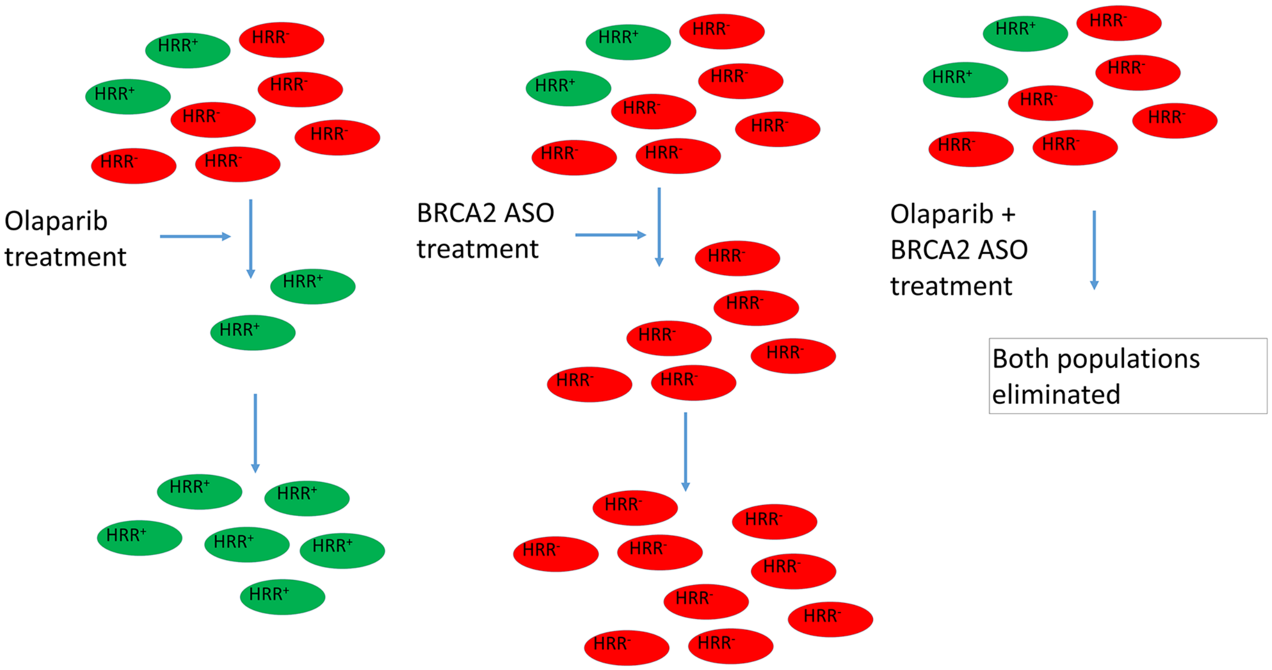

Supplementary Figure S1: Reciprocal positive selection for weakness. In a population heterogeneous for HRR-proficiency, BRCA2 ASO treatment will impair the growth of HRR-proficient cells and select for HRR-deficient cells. Olaparib treatment will accomplish the reciprocal of that by impairing the growth of HRR-deficient cells while selecting for HRR-proficient cells. Therefore, each treatment selects for cells that are susceptible to the other treatment. When BRCA2 ASO and olaparib treatment is combined, both HRR-proficient and HRR-deficient cells are affected, nullifying selection based on HRR-proficiency.

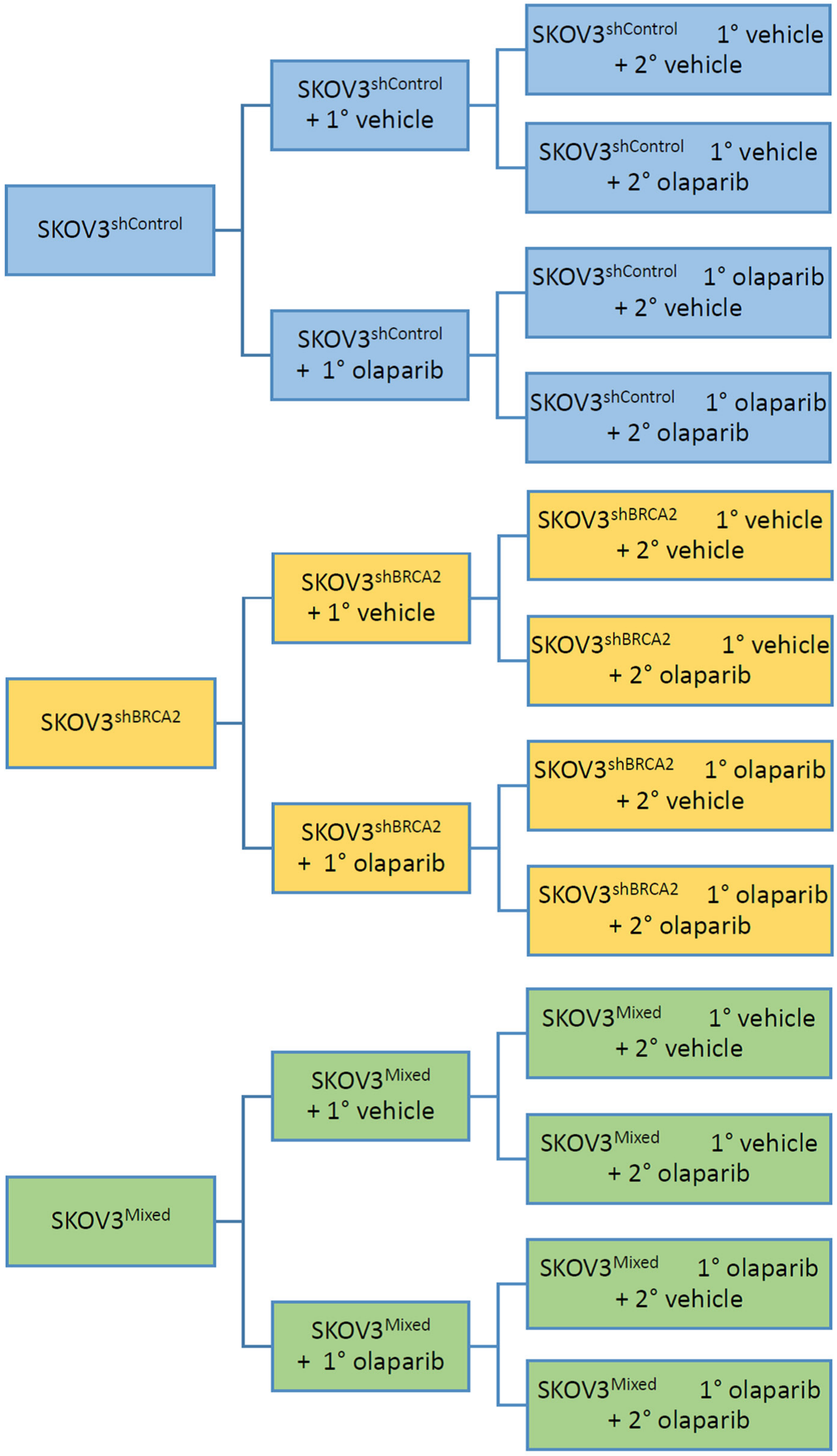

Supplementary Figure S2: Schematic for mixed cell experiments. The mixed cell population, as well as the unmixed populations were treated according to the experimental schematic. The experiment was conducted in a serial and continuous fashion, such that all treatment groups and cell populations were in culture for the same amount of time, and all controls were exposed to the same conditions.
